# A Wide Range of 3243A>G/tRNALeu(UUR) (MELAS) Mutation Loads May Segregate in Offspring through the Female Germline Bottleneck

**DOI:** 10.1371/journal.pone.0096663

**Published:** 2014-05-07

**Authors:** Francesco Pallotti, Giorgio Binelli, Raffaella Fabbri, Maria L. Valentino, Rossella Vicenti, Maria Macciocca, Sabina Cevoli, Agostino Baruzzi, Salvatore DiMauro, Valerio Carelli

**Affiliations:** 1 Department of Neurology, Columbia University, New York City, New York, United States of America; 2 Dipartimento di Scienze Chirurgiche e Morfologiche, University of Insubria, Varese, Italy; 3 Dipartimento di Scienze Teoriche e Applicate, University of Insubria, Varese, Italy; 4 Unità Operativa di Ginecologia e Fisiopatologia della Riproduzione Umana, Ospedale S.Orsola-Malpighi, University of Bologna, Bologna, Italy; 5 Dipartimento di Scienze Mediche e Chirurgiche (DIMEC), University of Bologna, Bologna, Italy; 6 IRCCS Istituto delle Scienze Neurologiche di Bologna, Ospedale Bellaria, Bologna, Italy; 7 Dipartimento di Scienze Biomediche e Neuromotorie (DIBINEM), University of Bologna, Bologna, Italy; Vanderbilt University Medical Center, United States of America

## Abstract

Segregation of mutant mtDNA in human tissues and through the germline is debated, with no consensus about the nature and size of the bottleneck hypothesized to explain rapid generational shifts in mutant loads. We investigated two maternal lineages with an apparently different inheritance pattern of the same pathogenic mtDNA 3243A>G/tRNA^Leu(UUR)^ (MELAS) mutation. We collected blood cells, muscle biopsies, urinary epithelium and hair follicles from 20 individuals, as well as oocytes and an ovarian biopsy from one female mutation carrier, all belonging to the two maternal lineages to assess mutant mtDNA load, and calculated the theoretical germline bottleneck size (number of segregating units). We also evaluated “mother-to-offspring” segregations from the literature, for which heteroplasmy assessment was available in at least three siblings besides the proband. Our results showed that mutation load was prevalent in skeletal muscle and urinary epithelium, whereas in blood cells there was an inverse correlation with age, as previously reported. The histoenzymatic staining of the ovarian biopsy failed to show any cytochrome-c-oxidase defective oocyte. Analysis of four oocytes and one offspring from the same unaffected mother of the first family showed intermediate heteroplasmic mutant loads (10% to 75%), whereas very skewed loads of mutant mtDNA (0% or 81%) were detected in five offspring of another unaffected mother from the second family. Bottleneck size was 89 segregating units for the first mother and 84 for the second. This was remarkably close to 88, the number of “segregating units” in the “mother-to-offspring” segregations retrieved from literature. In conclusion, a wide range of mutant loads may be found in offspring tissues and oocytes, resulting from a similar theoretical bottleneck size.

## Introduction

Human mitochondrial DNA (mtDNA) is assumed to be a clonal multi-copy genome of 16,5 kb that is strictly maternally inherited. In each cell, mtDNA may be present either as identical copies (homoplasmy) or as a mixed population of two or more different sequences (heteroplasmy or polyplasmy) [1]. Heteroplasmic mtDNA nucleotide changes, including those causing mitochondrial encephalomyopathies [2], segregate in tissues of the developing embryo as well as in germline cells. Somatic segregation of pathogenic mutations is relevant for clinical expression of mitochondrial diseases by affecting energy-dependent tissues that accumulate high, supra-threshold mutant loads [2], [3]. Germline segregation is crucial for maternal transmission of variable mutant loads to the offspring [3].

Heteroplasmy may be theoretically due to coexistence of individual organelles containing either exclusively mutant or exclusively wild-type genomes (inter-mitochondrial heteroplasmy) or to the coexistence in each mitochondrion of both mutant and wild-type genomes in different proportions (intra-mitochondrial heteroplasmy) [4]. The mtDNA molecules are associated with specific coating proteins in discrete nucleoids, physically attached to the inner mitochondrial membrane [5], which may themselves be either homoplasmic or heteroplasmic [6]. Admixture and complementation of heteroplasmic mtDNA genomes may be accomplished by mitochondrial fusion events and exchange of mtDNA between nucleoids [7]. Variable efficiency in complementation has been observed in cellular models harboring different mtDNA mutations [8], [9] but inter-mitochondrial complementation has been documented in a mito-mouse model carrying an mtDNA deletion [10]. Recent evidence suggests that nucleoids do not exchange genetic material frequently and are probably homoplasmic [11], [12], and may contain up to only one mtDNA molecule [13].

The load of mutant mtDNA may vary markedly between a mother and each of her children and a bottleneck mechanism has been postulated during the germline segregation of mutant mtDNA to explain rapid shifts of heteroplasmy observed within one generation [14]–[16]. However, the nature of the bottleneck mechanism in humans is still under intense debate. Recent studies led to several potential mechanisms that are not necessarily mutually exclusive. These include i) a marked reduction in the number of mtDNA molecules during the early stages of germline development [17]; ii) aggregation of identical segregating units without a reduction of mtDNA copy number, leading to rapid segregation due to sampling effect [18], [19]; iii) preferential replication of a subpopulation of genomes, implying an active selection [20]; iv) rapid mtDNA segregation in preimplantation embryos [21]. Most of the data collected so far have been obtained by studying animal models segregating clusters of mtDNA polymorphic variants [22]. The experimental models provided by animals carrying pathogenic mtDNA mutations (“mutator” mouse) suggested a purifying selection for the most severe mtDNA mutations [23], [24]. In humans, the bottleneck model has been tested only in a few studies, using both neutral polymorphisms and pathogenic mtDNA mutations segregating in relatively small pedigrees [25]–[30]. Apparently, different segregation patterns may operate depending on the mtDNA pathogenic mutation: the 8993T>G mutation associated with neuropathy, ataxia, retinitis pigmentosa (NARP) was characterized by skewed segregation in offspring or oocytes [27], whereas the 3243A>G/tRNA^Leu(UUR)^ mutation associated with mitochondrial encephalomyopathy, lactic acidosis, stroke-like episodes (MELAS) followed a random genetic drift model of segregation in a large sample of oocytes from a single woman [30].

We combined quantitative analysis of mtDNA heteroplasmy in both oocytes and somatic tissues to study the germline and somatic segregation of the 3243A>G/tRNA^Leu(UUR)^ pathogenic mutation [31] in two Italian pedigrees.

## Materials and Methods

### Patients

**Figure 1:**
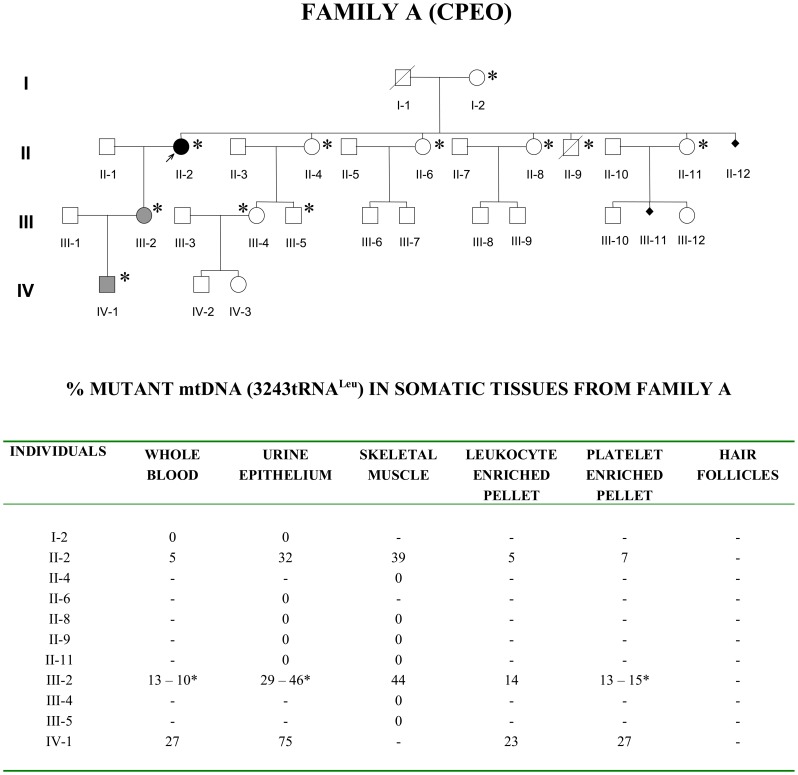
Pedigree of Family A. Filled symbol indicates the proband (II-2). Shaded symbols indicate asymptomatic individuals carrying the MELAS mutation. Asterisk indicate all the individuals who underwent molecular investigation. Individual III-2 underwent double samplings for some tissues (asterisk, in the table).

**Figure 2:**
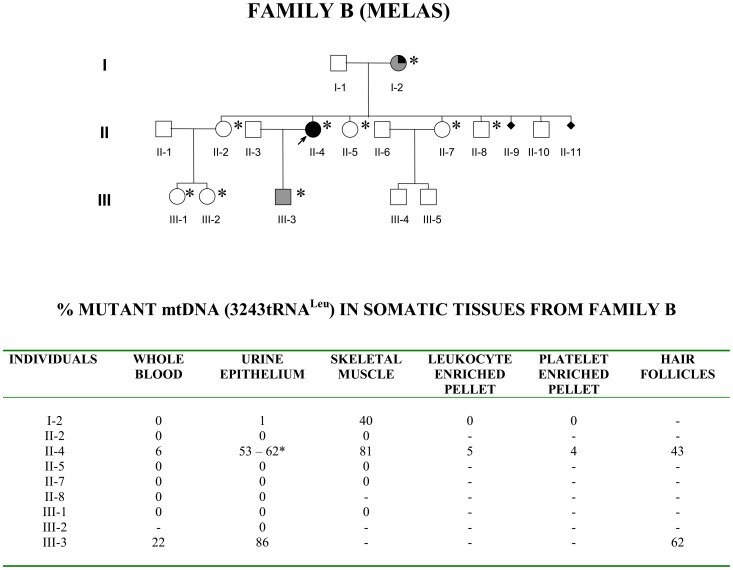
Pedigree of Family B. Filled symbol indicates the proband (II-4). Individual II-4 underwent double samplings for some tissues (asterisk, in the table).

We studied two previously reported [32] Italian maternal lineages (Family A in Figure 1 and Family B in Figure 2) carrying the heteroplasmic 3243A>G/tRNA^Leu(UUR)^ mutation. Briefly, the proband from Family A (II-2, Figure 1) was affected with chronic progressive external ophthalmoplegia (CPEO), whereas the proband from Family B (II-4, Figure 2) had the typical MELAS syndrome. Both probands had ragged-red-fibers (RRF) and/or cytochrome c oxidase (COX)-negative fibers in skeletal muscle with different mutation loads.

In family A, the proband’s only daughter (III-2) was asymptomatic excepted for frequent migraine attacks and she lacked RRF in muscle biopsy. Her son (IV-1) has been treated with growth hormone for short stature.

In family B, the proband’s mother (I-2) was clinically asymptomatic, whereas the proband’s only son (III-3) recently developed the full-blown MELAS syndrome.

After approval by the internal review board (1996–1998, Institute of Neurological Clinic, University of Bologna, Director Prof. Elio Lugaresi) and signed informed consent, 20 maternally related individuals from both families agreed to be enrolled in the study aimed at assessing the MELAS mutation loads in somatic tissues. In most cases, we have been able to collect blood samples (leukocytes- and platelet-enriched pellets or whole blood), muscle biopsies, urinary epithelium, and hair follicles. The proband’s only daughter in Family A (III-2), also underwent ovarian stimulation to allow collection of oocytes for genetic analysis and gave informed consent for an ovarian biopsy at the time of oocytes collection. Moreover, two of the proband’s sisters in Family B (II-5 and II-7) became pregnant during the time of our investigation and consented to prenatal diagnosis on cells recovered from amniotic fluid.

### mtDNA Analysis

Total DNA has been extracted by standard phenol/chlorophorm methods from somatic tissues, including amniocytes. Total oocyte DNA was recovered from single oocytes. Briefly, each oocyte was placed in an Eppendorf tube with one drop PBS and 1.5 µL proteinase K 10 mg/ml in ice, and centrifuged 30 sec 3000 rpm in an Eppendorf table-top centrifuge. After adding 50 µL sterile mineral oil, the mixture was centrifuged 30 sec as before, then digested at 37°C for one hour; digestion was blocked by boiling at 95°C for 15 minutes, followed by 80°C for 20 minutes. Each sample was then frozen and maintained at −80°C until the PCR amplification.

Heteroplasmy was determined by restriction fragment length polymorphism (RFLP) analysis after hot-last cycle PCR amplification as previously described [32]. The sensitivity of this method allowed detection of heteroplasmy as low as 1%.

### Estimation of Bottleneck Size in Our Families and Review of Previous Reports

We assessed the bottleneck size in two germline segregations from unaffected females carriers of the MELAS mutation to their offspring or oocytes. For the first segregation (Family A; female III-2 in Figure 1), we were able to estimate the proportion *p* of mutant mtDNA from the heteroplasmic load found in four primary oocytes collected from this woman and in the somatic tissues available from her only son. For the second segregation (Family B; female I-2 in Figure 2), the same estimate was obtained by averaging the loads of mutant mtDNA found in somatic tissues of five offspring. In both cases, *p* was taken as an estimate of *π*, the true proportion in the sample population. Under the binomial distribution, the variance was estimated by *p*(1–*p*)/*n*, whereas confidence intervals for *p* were estimated solving for the equation *z* = (*p*–*π*)/√*π* (1–*π*)/*n*. The binomial distribution applies if the levels of mutant mtDNA are solely determined by a sampling error such as may occur during a bottleneck. Confidence intervals were used, in both pedigrees, to test whether the mutant load in a given progeny was compatible with a random sampling event (i.e. the bottleneck in the mother).

The number of “units” undergoing the bottleneck was estimated according to equation (1) in Brown et al. [30] under the assumption that 24 cell divisions are needed to produce the full set of primary oocytes. Each segregating “unit” could be an mtDNA molecule or a nucleoid. We also applied the same statistical approach to a set of “mother-to-offspring” segregations reported in the literature, updating the series reviewed by Chinnery and colleagues [33], and evaluating tissue heteroplasmy in families in which there were at least three siblings besides the proband [34]–[39].

### Oocytes and Ovarian Biopsy

The proband’s unaffected daughter in Family A (III-2) underwent surgical laparoscopy during which oocytes were retrieved from both ovaries and a biopsy was taken from the right ovary. The oocytes were obtained after ovarian stimulation using a combination of a gonodotrophin-releasing hormone analogue (Triptoreline, Decapeptyl 3.75; Ipsen Biotec, Paris, France) and menotrophins (Metrodin HP, 75 IU; Serono, Milan, Italy) and immediately frozen in liquid nitrogen for DNA analysis [40].

The ovarian biopsy specimen was frozen in liquid nitrogen-cooled isopentane for histological and histoenzymatic staining, following the standard procedure used for muscle biopsies [41]. Ten µM sections were processed for hematoxylin/eosin standard staining and cytochrome c oxidase/succinate dehydrogenase (COX/SDH) double histoenzymatic staining. One age-matched control ovarian biopsy was used for comparison.

## Results

The heteroplasmic load of MELAS mutation assessed in various somatic tissues of maternally related individuals from Families A and B is summarized in Figures 1 and 2. The mutant mtDNA segregated only in some individuals along the maternal line of both families, as previously reported [32].

In Family A, the mutational event most likely occurred between individual I-2 and the CPEO proband (II-2 in Figure 1), considering that mutant mtDNA was absent in all other siblings of II-2, as well as in two maternal descendants in the third generation (individuals III-4 and III-5). We relied on the results in mtDNA from muscle and urinary epithelium, or at least one of the two tissues. The MELAS mutation was transmitted to the proband’s daughter, individual III-2 and to her son (IV-1), both currently unaffected. The mutant load slowly increased through these three generations, as shown by all tissues tested. In all individuals, the mutant loads in urinary epithelium and skeletal muscle were remarkably similar, whereas in blood-derived cells they were inversely correlated with age, as reported by others [42]–[44].

Four MII oocytes were retrieved from the proband’s daughter (III-2 in Figure 1). Under inverted microscope, the ooplasm had normal size and perivitelline space. Ultrastructurally, the oocytes had normally shaped nuclei with finely dispersed chromatin. The normal morphology of follicles and stromal cells was confirmed by ultrastructural analysis (data not shown).

**Figure 3:**
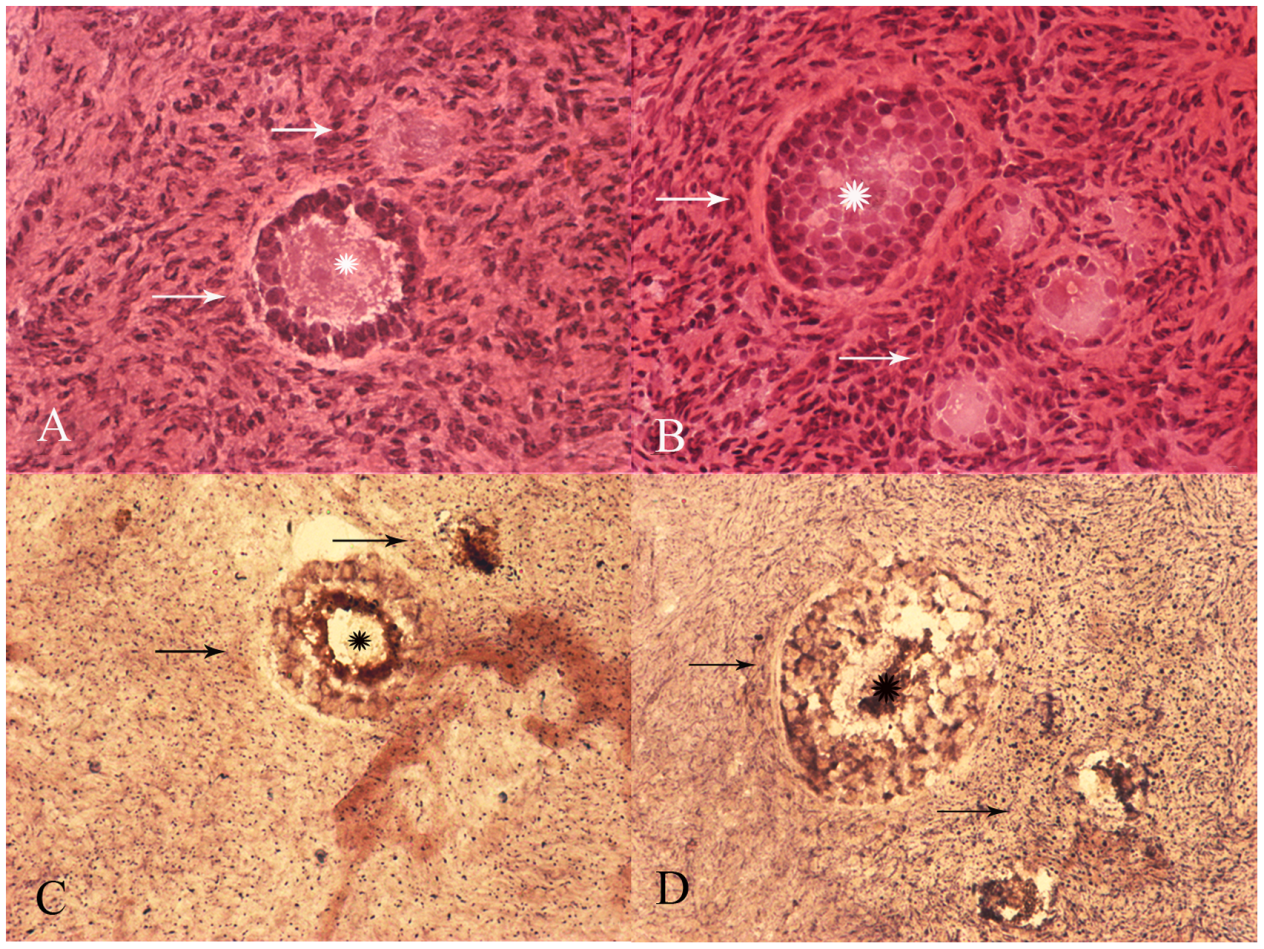
Ovarian follicles. A and C show two ovarian follicles (arrows) of individual III-2 (Family A), stained, respectively, with HE and COX/SDH; B and D, similarly, show three ovarian follicles (arrows), at different stages of maturation, of a control individual **(**magnification 20x). No evidence of reduced COX stain was observed in any of the tissues from the ovarian biopsy of the individual III-2, in particular the oocytes, as compared to the control (asterisks).

**Figure 4:**
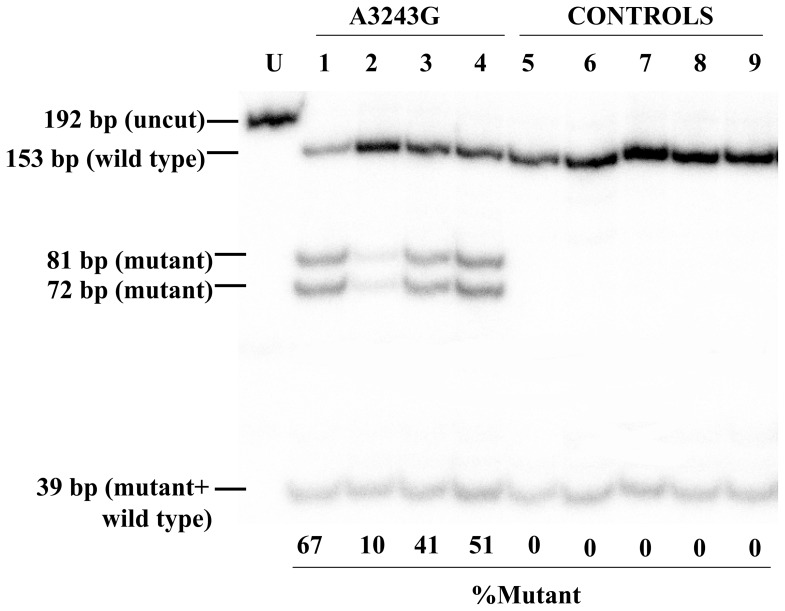
Quantification of the 3243A>G/tRNALeu mutation loads in four primary oocytes from individual III-2 (Family A) and from five control oocytes.

On double histoenzymatic staining for cytochrome-c-oxidase (COX) and succinate dehydrogenase (SDH) activities, oocytes within follicles were very intensely stained, whereas granulose cells had a less intense stain (Figure 3). This is compatible with the great amount of mtDNA copy number and mitochondria in oocytes. Remarkably, we failed to detect any sign of COX deficiency, neither in the oocyte cytoplasm nor in the other cell types (i.e.granulosa cells of the ovarian follicle and other stromal cells). Figure 4 shows the RFLP analysis in the four oocytes from individual III-2, which revealed mutant loads ranging from 10% to 67%.

In Family B, the female founder (I-2 in Figure 2) showed mutant mtDNA in skeletal muscle and urinary epithelium. This woman segregated mutant mtDNA only in one of her offspring, the proband affected with MELAS (II-4). None of the proband’s siblings had mutant mtDNA in any of the tissues investigated, nor did two maternal descendants in the third generation (III-1 and III-2). Furthermore, amniocytes collected during pregnancies of individuals II-5 and II-7 were also negative for the MELAS mutation (data not shown). Mutant mtDNA was transmitted from the proband to her only son, who is affected with MELAS like his mother. The tissue distribution pattern of somatic mutant loads was similar to that described for Family A, except that the female founder of this pedigree had undetectable mutant mtDNA in blood, only traces in urinary epithelium but a relatively high amount in skeletal muscle. Remarkably, this woman had had two miscarriages besides the five healthy offspring and the daughter with MELAS.

To investigate the “mother-to-offspring” germ line segregation of the MELAS mutation in these two maternal lineages (from individual III-2 in Family A and from individual I-2 in Family B), we estimated the percentage of mutant mtDNA in somatic tissues of each offspring in Families A and B, and in each oocyte in Family A. The germline segregation was compatible with a bottleneck event in the mother, according to the binomial distribution. Thus, different mutation loads in the progeny have to be ascribed to chance alone. The bottleneck size, based on the assumption that 24 cell divisions are needed to produce primary oocytes, consisted of 89 segregating units for Family A and 84 for Family B, if we consider only the mutant load in skeletal muscle (Table 1). If we take into account the mtDNA heteroplasmy of urinary epithelium in both Family A and B, the segregation units were 108 (Family A, oocytes from subject III-2 plus urinary epithelium from the only son) and 110 (Family B, urinary epithelium from all offspring) (Table 1).

**Table 1:** Estimate of bottleneck sizes for the MELAS mutation from a review of the literature.

Source	Ref.	Clinical phenotype mother	Clinical phenotype offspring	Proband’s phenotype	Tissue analyzed	p	C.I.	N
Martinuzzi et al (1992)	34	Unaffected	Four, unaffected	MELAS	Skeletal muscle	0.693	0.262–0.935	84
Liou et al (1994)	35	Headache, episodic vomiting	Four, one MELAS	MELAS	Hair follicles	0.237	0.030–0.740	120
De Vries et al (1994)	36	Unaffected	Five, two MELAS and one deafness	MELAS	Fibroblasts	0.282	0.065–0.688	108
De Vries et al (1994)	36	Unaffected	Four, two MELAS and one deafness	MELAS	Skeletal muscle	0.500	0.150–0.850	83
Huang et al (1996)	37	Unaffected	Four, one MELAS	MELAS	Skeletal muscle	0.175	0.024–0.644	84
Koga et al (2000)	38	Muscle weakness	Four, one Leigh syndrome	Leigh syndrome	Hair follicles	0.548	0.175–0.873	84
Dubeau et al (2000)	39	Deafness, cardio-myopathy, short stature	Eight, two MELAS and one migraine and ptosis	MELAS	Urinary epithelium	0.283	0.082–0.443	59
Family A (this work)	–	Unaffected	Four oocytes	CPEO	Oocytes	0.423	0.113–0.808	89
Family A (this work)	–	Unaffected	Four oocytes+one son	CPEO	Oocytes+urinary epithelium from IV-1	0.488	0.188–0.793	108
Family B (this work)	–	Unaffected	Four, one MELAS	MELAS	Skeletal muscle	0.203	0.031–0.665	84
Family B (this work)	–	Unaffected	Five, one MELAS	MELAS	Urinary epithelium	0.115	0.014–0.551	110

p = frequency of mutant mtDNA in the progeny; C.I. = lower and upper limits of confidence interval for π at the 0.95 level; N = estimated size of the bottleneck.

We reviewed previously reported families segregating the MELAS mutation [33]–[39] and selected those in which the *p* of mutant mtDNA was reported for both mother and progeny and included, besides the proband, at least three siblings. We then subjected these “mother-to-offspring” segregations retrieved from the literature to the same test for the binomial distribution that we have used for the analysis of our Italian families. In all included cases (see Table I) the *p* of mutant mtDNA in the progeny was compatible with a random segregation event in the mother. The number *N* of “segregating units” was in the range of 59–120, with an average number of *N* = 88 (confidence interval at the 0.95 level was 75≤ *N* ≤101), remarkably close to the values estimated in our study, *N* = 89 for Family A and *N* = 84 for Family B. These segregations were calculated using different somatic tissues, such as skeletal muscle, hair follicles, fibroblasts and urinary epithelium (Table 1).

**Figure 5:**
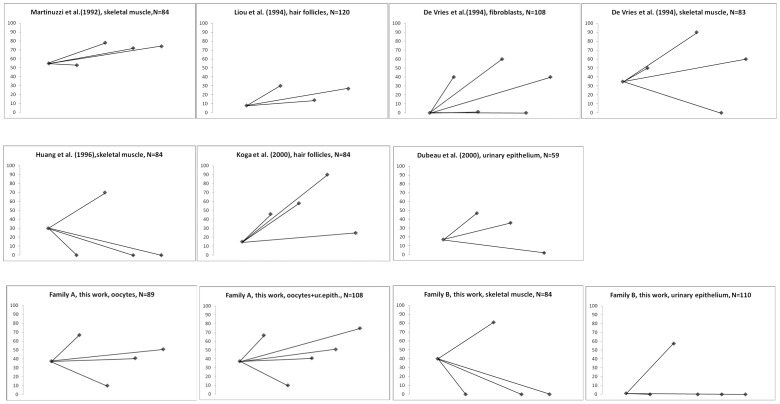
Graphical representation of mother-to-offspring transmission of the MELAS mutation in the two Italian families and the seven other pedigrees retrieved from literature (see Table 1). The mutant load of MELAS mutation (%) is on the y axis. In all panels, the leftmost point is the mother’s mutant load, connected to each of the offspring mutant load. The reference, the tissues from which mtDNA mutant load has been assessed and the bottleneck size are indicated.

Overall, these cumulative data show a close relationship between the tissues analyzed and the relative calculation for bottleneck size (N): for both a postmitotic tissue, such as skeletal muscle and oocytes, N resulted similar, despite the resulting mutation load in offspring was largely distributed in Family A and skewed to the extremes in Family B. Our literature revision revealed that in most cases the “mother-to-offspring” transmission resembled Family A [34], [35], [36], [38], [39], whereas only one family was essentially identical to Family B [37], still with very similar estimated bottleneck sizes. The overview of the relationship between mother and offspring mutant loads from our Families A and B, and those retrieved from literature are graphically represented in Figure 5, including the theoretical bottleneck calculated for each of these segregations.

## Discussion

This study shows that germline segregation of the 3243A>G/tRNA^Leu^ MELAS mutation may lead to a wide range of mutational loads in offspring through a similar bottleneck size. Its estimation in the two Italian families here investigated was remarkably close to the average number of segregating units calculated for other “mother-to-offspring” germline segregations retrieved from the literature. In Family A, individual III-2 transmitted intermediate, largely distributed loads of heteroplasmic mutant mtDNA (10% to 75% mutant; Figure 5), as measured in four of her oocytes and in her only son. This resembled most of the other segregations retrieved from the literature (Figure 5). In contrast, in Family B we observed a sharply skewed transmission of mutant from individual I-2 to only one of her offspring (81% mutant; Figure 5). All other siblings had only wild-type mtDNA in the tissues analyzed (0% mutant; Figure 5), including amniocytes from two pregnancies of individuals II-5 and II-7. This was paralleled by only one family previously reported by Huang et al. [37], which had an essentially identical distribution of mutant loads in skeletal muscle of offspring (Figure 5).

The number of “segregating units” (bottleneck size), calculated in these two Italian families and in the several cases retrieved from the literature [28]–[30] was substantially lower than the 173 segregating units estimated by Brown et al. in the only study that sampled a large set of oocytes (N = 82) from a female carrier of the same MELAS mutation [30]. An important limitation of the current study and those retrieved from the literature is the large error associated with the variance estimated from a very low sample number (≥4) [45]. This is an obvious drawback by working with living patients from human pedigrees. A recent study [46] on the segregation of the MELAS mutation through the human embryofetal development concluded that random drift drives germline segregation, similar to Brown’s and colleagues conclusions [30], but with some appreciable individual-dependent differences in bottleneck size. Interestingly, in a study based on a large cohort of individuals carrying the MELAS mutation, the mothers with a mutation load greater that 50% tended to have offspring with lower or equal heteroplasmy, whereas the opposite was true for mothers with less than or equal to 50% mutation load [47]. These authors concluded that the random genetic drift model could not fully explain the transmission of the MELAS mutation [47]. Ascertainment bias has also to be considered. The recent finding that one in 200 healthy humans harbors a pathogenic mtDNA mutation out of the ten most frequent, indicates that there is a large pool of maternal lineages were probably these mutations segregate silently, and are possibly selected out, missing to express any pathology and not being ascertained at all [48]. Thus, investigation of single pedigrees identified by an affected proband introduces a bias that may be resolved by pooling large cohort of families through multi-centric studies, or by meta-analyses of reported pedigrees.

The analysis of somatic segregation of the MELAS mutation in our two families confirmed that the mutant load is inversely correlated with age in blood cells, whereas skeletal muscle is the tissue of choice, followed by urinary epithelial cells, for detection of the mutation [42]–[44]. The pattern of mutational load in somatic tissues distinguished the two families, which also differed for the clinical phenotype. In Family A (CPEO), the mutational load in the unaffected female individual III-2 appeared to be similar in skeletal muscle (44%) and urinary epithelium (29%–46%), whereas in the female individual I-2 of Family B (MELAS) the mutational load in skeletal muscle (40%) was much higher than in urinary epithelium (1%). This latter observation might be related to the skewed transmission of mutant mtDNA in the offspring of this woman, resulting in one MELAS patient (81% mutant mtDNAs in skeletal muscle), two miscarriages conceivably due to very high mutant loads, and all remaining unaffected individuals with wild-type mtDNA.

Many recent studies have tried to tackle the issue of mtDNA germline segregation testing the bottleneck hypothesis [17]–[22]. These studies have employed murine and primate heteroplasmic models and there is no consensus on whether the bottleneck exists, whether there are one or more bottlenecks, and at what stage of development the bottleneck(s) operate. These models do not closely recapitulate the situation of a single mtDNA pathogenic mutation segregating along the maternal line of human pedigrees because most heteroplasmic animals were generated by mixing two mitochondrial genomes that differed for a cluster of polymorphic variants, which may have no or small functional relevance [49]. This condition is different from the case of a single pathogenic mtDNA mutation arising on a clonal mitochondrial genome, which is typical of humans with mitochondrial disorders. Important differences between the two situations may include the nucleoid composition and the level of mtDNA exchange, if any, between nucleoids. Nucleoids seem to follow the faithful replication model, without consistent genome exchange [6], [11], [13]. Furthermore, it has been demonstrated that mtDNA molecules may recombine within mitochondria [50]–[52], a phenomenon that is not relevant when mtDNA is clonal as in most humans, but that may become important in the case of different coexisting genomes with clusters of distinct variants, as in the heteroplasmic animal models or sometimes in humans with multiple heteroplasmies [17]–[21], [53]. No studies address how frequently mtDNA recombination may occur, in which cell type, or during which stage of germ line segregation. Neither heteroplasmic animal models [17]–[21], [49] nor the few available pathologic mito-mouse models [10], [23], [24] have been fully exploited yet to answer all these questions.

One final question concerns the possible selective pressure on mtDNA pathogenic mutations. The currently available mito-mice clearly indicated that severe mtDNA mutations undergo purifying selection over a few generations [23], [24]. The segregation of the MELAS mutation in human tissues has been proposed to be non-random [54], and in vitro studies using cybrids with different nuclear backgrounds showed that segregation of the mutant mtDNA could be driven in opposite directions depending on the nuclear genome [55]–[57]. Thus, selection of mutant mtDNA may occur differently in different somatic tissues, impinging on the phenotypic expression. Whether such a genotypic selection may also operate during the germ line segregation for “mild” changes, including the MELAS mutation, is currently debated, casting doubts on the random genetic drift mechanism [47]. Staining the ovarian tissue for the histoenzymatic COX/SDH activities failed to reveal any COX-deficient oocyte, nor other cell types. This may indicate that in this particular case there was no oocyte with supra-threshold loads of MELAS mutation or that a very efficient complementation occurs within oocytes, which may escape in the case of MELAS mutation any selection along the germline.

In conclusion, the mechanisms governing the germline segregation and the subsequent somatic distribution of single pathogenic mtDNA mutations in humans remain far from being elucidated. Our study of mother-to-oocytes/offspring tissues transmission of the same pathogenic MELAS mutation shows how wide may be the range of mutant loads segregating through the same bottleneck size.
