# Correction: A Wide Range of 3243A>G/tRNALeu(UUR) (MELAS) Mutation Loads May Segregate in Offspring through the Female Germline Bottleneck

**DOI:** 10.1371/journal.pone.0115961

**Published:** 2014-12-12

**Authors:** The PLOS ONE Staff

There is an error in the affiliations for the seventh author. Sabine Cevoli should not be affiliated with Affiliation 7, Dipartimento di Scienze Biomediche e Neuromotorie (DIBINEM), University of Bologna, Bologna, Italy. The full, correct list of authors and affiliations is given below.

Francesco Pallotti^1,2^, Giorgio Binelli^3^, Raffaella Fabbri^4,5^, Maria L. Valentino^6,7^, Rossella Vicenti^4,5^, Maria Macciocca^4,5^, Sabina Cevoli^6^, Agostino Baruzzi^6,7^, Salvatore DiMauro^1^, Valerio Carelli^6,7*^

**1** Department of Neurology, Columbia University, New York City, New York, United States of America

**2** Dipartimento di Scienze Chirurgiche e Morfologiche, University of Insubria, Varese, Italy

**3** Dipartimento di Scienze Teoriche e Applicate, University of Insubria, Varese, Italy

**4** Unità Operativa di Ginecologia e Fisiopatologia della Riproduzione Umana, Ospedale S.Orsola-Malpighi, University of Bologna, Bologna, Italy

**5** Dipartimento di Scienze Mediche e Chirurgiche (DIMEC), University of Bologna, Bologna, Italy

**6** IRCCS Istituto delle Scienze Neurologiche di Bologna, Ospedale Bellaria, Bologna, Italy

**7** Dipartimento di Scienze Biomediche e Neuromotorie (DIBINEM), University of Bologna, Bologna, Italy

* E-mail: valerio.carelli@unibo.it
